# Research on strategies for enhancing drug knowledge dissemination on Chinese social media WeChat public accounts based on text mining technology

**DOI:** 10.3389/fphar.2025.1569863

**Published:** 2025-08-26

**Authors:** Xihui Yu, Xiaotong Chen, Xia Yan, Xuejun Wu, Yizhi Zhang, Xiajiong Luo, Weihao Ma, Hongbo Fu, Yaofeng Zhang

**Affiliations:** Department of Pharmacy, The Second Affiliated Hospital of Shantou University Medical College, Shantou, Guangdong, China

**Keywords:** natural language processing, topic modelling, term frequency-inverse document frequency (TF-IDF), VOSviewer, WeChat, visualization analysis, pharmic science popularization, medication adherence

## Abstract

**Objective:**

Health science popularization is an important means to improve public health literacy, promote healthy lifestyles, prevent diseases and respond to health crises, which is of great significance for improving the overall health of the people. Strengthening the medication education of patients is also one of the key factors to improve patients’ medication adherence. In order to strengthen the dissemination of pharmaceutical popular science articles and give full play to the value of pharmaceutical popular science, this study takes WeChat public account as the research platform to explore effective strategies to improve pageviews of science popularization. It provides references for science popularization workers, so that science popularization can play a better role in improving the public’s knowledge of medication safety.

**Methods:**

Taking the well-known pharmaceutical science popularization WeChat account “PSM Medicine Shield Public Welfare” as an example, we combined the Term Frequency-Inverse Document Frequency (TF-IDF) algorithm and VOSviewer visualization analysis technology to construct a hot topic analysis model for pharmaceutical science popularization articles, and analyzed the common rules and characteristics of successful hot articles. Latent Dirichlet Allocation (LDA) and The Bidirectional Encoder Representations from Transformers Topic (BERTopic) model were used to realize the construction of the topic model.

**Results:**

The model selected the top 20% of popularization articles with the greatest reading volume between 2015 and 2023 as the database for text mining. The clustering results indicated that the public was interested in these five types of pharmaceutical science popularization themes: drug dosage, drug side effects, children’s infections, the efficacy of traditional Chinese medicine and Chinese patent medicines, and the usage methods of different drug administration routes. The public’s interest in topics changed from drug side effects to practical drug usage issues, as seen by the keyword time series graph.

**Conclusion:**

Pharmaceutical professionals may more effectively discover hot themes in the industry by combining the TF-IDF algorithm with VOSviewer visualization analysis and LDA and BERTopic in the text mining. This improves the readability of popularization articles and the impact of WeChat accounts, which may improve medication adherence and raise public awareness of medication usage.

## 1 Introduction

Medication non-adherence is still a major and ongoing problem that has a big impact on patient outcomes and the long-term viability of healthcare systems. One of the key patient-centered elements in medication adherence is the patient’s awareness of their condition and medications (Kardas et al., 2024). Health science popularization through social media platforms has become a popular online educational method for safe medication. With government’s proposal of the Healthy China Strategy, universal health has become the focus of increasing attention in China (Zhuang et al., 2022). The popularization of digital health science is a potent addition to offline health science popularization, which is very effective in promoting public services, leveraging new communications, and tracking trending topic of social concern. Because popularized articles addressed to a lay audience lead laypeople to agree more with the knowledge than scientific articles addressed to expert audiences do (Scharrer et al., 2017), digital health science popularization is widely used for education of safe medication. In China, hospital pharmacy services are currently in a transitional development stage, with a shift from a focus on ensuring drug supply to providing patient-centered pharmaceutical technical services. Against the backdrop of rapid development in Internet information technology, the State Council has successively issued multiple policies to encourage medical institutions to promote the “Internet plus healthcare” online service model (Yang et al., 2021). For instance, the “Opinions of the State Council on Implementing the Healthy China Initiative”, the “Notice of the State Council on Issuing the Outline of the Healthy China 2030 Plan”, and the “Healthy China Initiative (2019–2030)” have all put forward new requirements for “promoting Internet plus precise health science popularization” (Jiang and Jiang, 2021). Under the continuous deepening of medical reform, pharmacy personnel are exploring new pharmacy service models with the aid of information technology.

Conducting pharmacy services through the WeChat official account platform not only enhances the credibility of hospital pharmacists and showcases the brand characteristics of the pharmacy department, but also serves as a powerful supplement to offline pharmacy services. Its dissemination features are conducive to expanding the coverage of pharmacy services. As a carrier for pharmacy science popularization and communication, the WeChat official account can not only publicize relevant national medical policies and promote their implementation and application, but also disseminate knowledge on rational and safe medication, thereby improving patients’ medication compliance and reducing the risk of medication errors. Currently, the majority of medical institutions and pharmacy service groups use WeChat official accounts to conduct online science popularization and education for pharmacy services. However, there are also the following problems in the operation of WeChat official accounts: First, the operators of the official accounts usually manage them part-time during their rest time. Due to the lack of professional capabilities in material collection, content planning, and article production, the quality of operation may decline or even fail to sustain long-term operation. Second, the content of the official accounts is monotonous. For example, the content is limited to the journal content and lacks feedback data from users for improvement, resulting in insufficient innovation and low user attention. It is difficult to enhance its dissemination influence and establish a strong connection between the official account and users. Third, the topics and focus of health science popularization are highly specialized, but the target audience of the official accounts has limited professional knowledge reserves, leading to poor affinity and readability of the health science popularization. Fourth, the content of health science popularization lacking practicality and simplicity fails to meet the needs of multiple reading groups, which is not conducive to the dissemination of pharmacy education content. With the development of the Internet and the explosive growth of information, there are still many challenging tasks for pharmacists to improve pharmaceutical care through the public number platform, such as quickly obtaining effective information from the platforms with a huge amount of complex information, improving the utilization rate of high-quality articles of health science popularization, enhancing the readability of pharmic science popularization and the influence of public accounts.

Currently, unlike in the case of academic papers where keywords are conveniently provided, the articles on WeChat public accounts do not offer such a feature. This makes it impossible for us to quickly grasp the main topic of the article. As a result, the conventional reading method will not facilitate the efficient capture of hot topics by the science popularization worker. Natural language processing (NLP) has become an essential tool for extracting meaningful insights from vast amounts of biomedical literature (Ma et al., 2025). Topic modeling has emerged as a foundational technique within the domain of NLP and text mining, providing an essential methodology for extracting insightful patterns from extensive and intricate text datasets. Traditional topic modeling methods such as Latent Dirichlet Allocation (LDA) have established a robust framework for understanding and organizing unstructured text data. The Bidirectional Encoder Representations from Transformers Topic (BERTopic) model is another advanced NLP tool that can analyze large quantities of textual data from medical journal (Baird et al., 2025). Studies comparing LDA and BERT have demonstrated that BERT outperforms LDA in extracting deeper and distinct topics, identifying intricate topic relationships in datasets with complex linguistic structures (Egger and Yu, 2022).

Topic modelling, a method capable of extracting abstract topics from documents, is commonly used to identify key concepts in texts. Besides the widely used LDA method, the application of BERTopic, a deep learning-based model, is becoming increasingly popular. However, this approach is often applied to scientific literature and social media content, with limited research on pharmic science popularization texts. Term Frequency-Inverse Document Frequency (TF-IDF) was first proposed by Salton in 1973 and is a commonly used weighting technique in information retrieval and text mining (Kwon et al., 2018). Firstly, this study uses the visualization analysis software VOSviewer as an auxiliary tool to obtain the clustering and time series views of keywords based on the TF-IDF algorithm. Secondly, we realized the construction of the topic model of LDA and BERTopic in the pharmic science popularization texts. By applying these two NLP models to this specific dataset, we evaluated their effectiveness and accuracies in their ability to handle the complexities inherent in pharmic science popularization texts. This study aims to effectively explore the characteristics of highly-read pharmic science popularization articles, summarize the experience of writing, and provide certain references for promoting the development of pharmic science popularization through NLP technology.

## 2 Materials and methods

### 2.1 Research object

PSM Medicine Shield Public Welfare is a non-profit organization jointly initiated and established by the China Association of Nonprescription Drugs, the Chinese Pharmaceutical Association, and the Chinese Medical Association. The public account of “PSM Medicine Shield Public Welfare” updates science popularization articles every day, which is widely clicked and read by pharmacists. It is representative among public accounts on pharmic science popularization. Therefore, this study takes the public account as the research object and collects all the science popularization articles which published from 2015 to 2023.

### 2.2 Text visualization analysis method

#### 2.2.1 Text extraction and preprocessing

This study used public account data analysis platform (https://www.cimidata.com/) to collect and statistically analyze the titles, publication times, and reading volumes of all popular science articles published by the PSM Medicine Shield Public Welfare public account from 2015 to 2023. The statistical data is collected on 17 June 2024. In this study, based on the Pareto Principle, we rank the reading volumes of the collected popular science articles from high to low and define the top 20% of articles as articles with a high number of pageviews (Timmis et al., 2023). Then, we organized the full text of articles with high pageviews. The title, publication time, organization also were collected in Excel.

The core component of text mining, which seeks to standardize and clean the corpus, is text preprocessing. Screening procedures, stop-word removal, word segmentation, and part-of-speech tagging are often included. Chinese text preprocessing differs from English text preprocessing in that it does not need stem analysis, citation, or case normalization. Data screening, stop word removal, domain dictionary construction, and word segmentation are the four substeps that are designed.

Data screening is the initial stage. Text normalization is necessary before Chinese word segmentation, which is often accomplished using regular expressions, due to the unpredictability of text data sets and the prevalence of professional words and idioms. This makes it possible to combine word groupings that have distinct expressions but the same meaning.

Eliminating stop words is the second stage. Prepositions, pronouns, conjunctions, and other punctuation that are often used in a text but have no practical significance. They are referred to as stop words, which are not useful for analyzing the text’s topic. By importing the list of stop words, these pointless words may be eliminated. The list of stop words is available for download from Baidu input methods and the Modern Chinese Function Words Dictionary.

Creating a domain dictionary is the third stage. The way is described varies greatly since human language is diverse. To improve text segmentation, a specific domain dictionary must be built beforehand. The custom dictionary comes from the drug names in the National Drug Code Base issued by the National Medical Products Administration. The purpose of the custom dictionary is to enable the word segmentation tool to recognize professional drug names.

The segmentation of words is the fourth phase. By locating the term boundary, the corpus is decomposed into discrete and linguistically meaningful terms (Miner et al., 2012). Chinese word segmentation follows certain guidelines to reassemble continuous Chinese sentences into word sequences. By removing stop words, eliminating the interference of meaningless words, and further introducing the constructed domain dictionary, word segmentation can be carried out directly.

All the text preprocessing above was accomplished through Data-Information-Knowledge-Wisdom (DIKW) 10.2 software (XueShuDianDi, 2025). The above steps do not require any parameter settings. They can be accomplished by simply following the corresponding modules.

#### 2.2.2 Feature selection

After the above data cleaning and word segmentation, we need to extract keywords from each text. TF-IDF is currently the most frequently used feature weighting algorithm. It assesses the importance of a word to a text by calculating the weight of the word in the text. TF-IDF has two meanings: one is “term frequency” (Term Frequency, abbreviated as TF), and the other is “inverse document frequency” (Inverse Document Frequency, abbreviated as IDF). The TF-IDF value is equal to the product of term frequency and inverse document frequency. The larger the TF-IDF value of a word in an article, the more frequently it appears in that text but is rare in other texts, and it can be considered a representative word of that text. In this study, we use the TF-IDF algorithm to count the high-frequency words in the effective data text.

The text’s feature dimension remains quite high even after feature vectorization and text preprocessing. In order to reduce the computational complexity, feature extraction is needed. The purpose to extract words is that can represent the document from the text for summarizing the content of the popular science articles. Feature extraction is a dimensionality reduction approach that calculates a feature’s score value according to a feature evaluation function, arranges these features according to the score results, and selects features with high score values as feature items. It minimizes the number of characteristics and the computational complexity of modeling and enhances clustering efficiency (Al Dweik et al., 2017).

As a typical feature selection approach, the Term Frequency Inverse Document Frequency (TF-IDF) is commonly utilized as a feature evaluation function for feature extraction (Onan, 2021). TF-IDF is currently the most frequently used feature weighting algorithm. It assesses the importance of a word to a text by calculating the weight of the word in the text. The TF of keywords is given as Formula 1. In Formula 1, *n*_*i,j*_ denotes the number of instances of the keyword *t*_*i*_ that occurs in the science popularization document *d*_*j*_ and ∑_*K*_ *n*_*k,j*_ is the number of all keywords in the science popularization document *d*_*j*_. The IDF of keywords is expressed as Formula 2. In Formula 2, |D| represents the total number of science popularization documents and |{*j*: *t*_*i*_ ∈ *d*_*j*_}| is the number of documents containing keyword *t*_*i*_ to avoid this item being zero and the divisor being zero, and it is generally expressed as 1+|{*j*: *t*_*i*_ ∈ *d*_*j*_}|. IDF indicates that the less times a keyword occurs in an accident record document, the higher the weight assigned to the keyword. It is the reverse of TF, but it is susceptible to unusual keywords. The TF-IDF value is equal to the product of term frequency and inverse document frequency (Formula 3). The larger the TF-IDF value of a word in an article, the more frequently it appears in that text but is rare in other texts, and it can be considered a representative word of that text.

$${tf}_{i,j}=\frac{n_{i,j}}{\sum_{K}n_{k,j}}$$ (1)

$${idf}_{i}=\log\frac{\left|D\right|}{1+\left|\left\{j:t_{i}\in d_{j}\right\}\right|}$$ (2)

$${TF-IDF}_{i,j}={tf}_{i,j}\times {idf}_{i}$$ (3)

#### 2.2.3 Text visualization

Word cloud is a visualization technique for text information, which can intuitively display the frequency of keywords. The font size of each word indicates its relevance (Nalon et al., 2020). We calculated the top 50 TF-IDF values of each popular science article with high pageviews using the TF-IDF algorithm and counted the frequency of each word. We used Python 3.10 to create word clouds for these keywords and their frequencies. TF-IDF values were calculated by DIKW 10.2 software. In terms of TF-IDF parameter settings, the number of keywords to be extracted is set to 50.

Nees Jan van Eck developed the bibliometric tool VOSviewer based on VOS visualization technology, which can be used to analyze the development status of a certain discipline or field in an intuitive and visual form (van Eck et al., 2010). In this study, we initially screened out keywords using the TF-IDF algorithm, and then referred to the way of importing literature in VOSviewer to import the text and analyze the co-occurrence and coupling relationships of them. We used a visualization chart to review the theme characteristics and development trends of the high-reading popular science articles on drug science of this public account from 2015 to 2023. The parameter settings for VOSviewer are as follows. Method: Association strength. Attraction: 2. Repulsion: 1. Resolution: 1.00. Min. cluster size: 1.

#### 2.2.4 Determination of the number of topics

The clustering algorithm employs K-means. K-means algorithm is a basic widely utilized clustering method in both academia and industry, valued for its simplicity and efficiency (Liu et al., 2023). The optimal cluster number was confirmed by Silhouette coefficient, Calinski-Harabasz index and Davies-Bouldin index through DIKW 10.2 software (Prompook et al., 2025).

The Silhouette coefficient (SC) is a method for measuring the quality of data clustering. It assesses how similar each data point is to its cluster compared to the nearest neighboring cluster. The SC is calculated by averaging the distance between a point and other points in the same cluster, and the average distance to the points in the nearest cluster. The values of SC range from −1 to 1, where values closer to 1 indicate that data points are consistent within their cluster and distinct from other clusters.

The Calinski–Harabasz index (CHI) is a clustering measure used for evaluating the quality of data clustering. It is fast to compute for large datasets and is suitable for evaluating multiple clusters. The CHI score is calculated based on the ratio between the between-cluster dispersion and the within-cluster dispersion. A higher CHI score indicates better clustering of data segments.

The Davies-Bouldin index (DBI) is a widely used method for evaluating clustering and determining the most suitable number of clusters. It is calculated based on the ratio of intra-cluster dispersion to inter-cluster dispersion, considering the quality and proximity of the cluster data. A lower DBI value indicates better clustering quality, implying that the clusters have low within-group dispersion and high between-group differences.

#### 2.2.5 Topic modelling

LDA is an unsupervised probabilistic topic modeling method. The model assumes that each document is composed of a mixture of multiple latent topics, and each topic is represented by a probability distribution of words. The process of solving the LDA model using DIKW 10.2 software: (1) Reading data: for example, reading raw data from an Excel file. (2) Data preprocessing: this process includes for word segmentation, removing stop words, replacing synonyms, deleting unnecessary spaces, etc. (3) LDA modeling: this part of the operation includes word frequency vectorization processing, finding the optimal number of topics, modeling the optimal number of topics, outputting the results, and outputting various drawings obtained after LDA calculation. The threshold for word frequency extraction is set at 2. The number of clusters’ themes is set at 4. The number of words for each topic is set at 20. We chose TF-IDF method instead of word frequency to obtain the topic words.

BERTopic assigns documents to topics probabilistically, identifying frequent and specific representative terms. The process of solving the BERTopic model using DIKW 10.2 software. The primary workflow steps included: (1) preprocessing data sets for embedding preparation, (2) embedding textual data into multidimensional vectors to capture contextual nuances, (3) count vectorization converting text into token frequency matrices, (4) dimensionality reduction of embedded data to facilitate clustering, (5) clustering to detect dense regions within reduced-dimensional space and (6) custom naming of topics.

## 3 Results

### 3.1 Release time, quantity and regional distribution of popular science articles on drug science

A total of 6,457 popular science articles from PSM Drug Shield Public Welfare from 2015 to 2023 were collected in this study. According to the Pareto Principle, we screened out the top 20% of popular science articles in terms of pageviews. After reading, cleaning and classification, the top 1,264 science popularization texts in terms of pageviews were included in the research objects.

**FIGURE 1:**
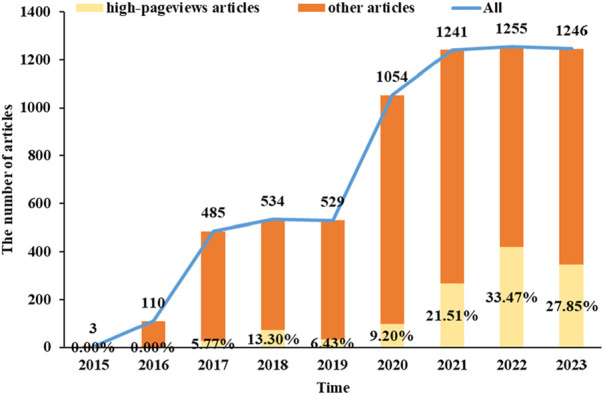
The statistical distribution map of published time and number of popular science articles on PSM Medicine Shield Public Welfare in 2015–2023.

The release situation of popular science articles on drug science of PSM Drug Shield Public Welfare from 2015 to 2023 is shown in Figure 1. The numbers at the top of the bars represent the total number of popular science articles published by the public account in each year, and the yellow parts represent the distribution and proportion of high-reading popular science articles in each year.

**FIGURE 2:**
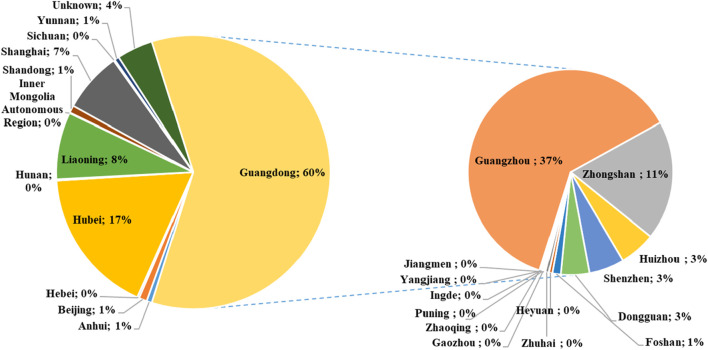
Regional distribution map of high-pageviews science popularization articles in 2015–2023.

**FIGURE 3:**
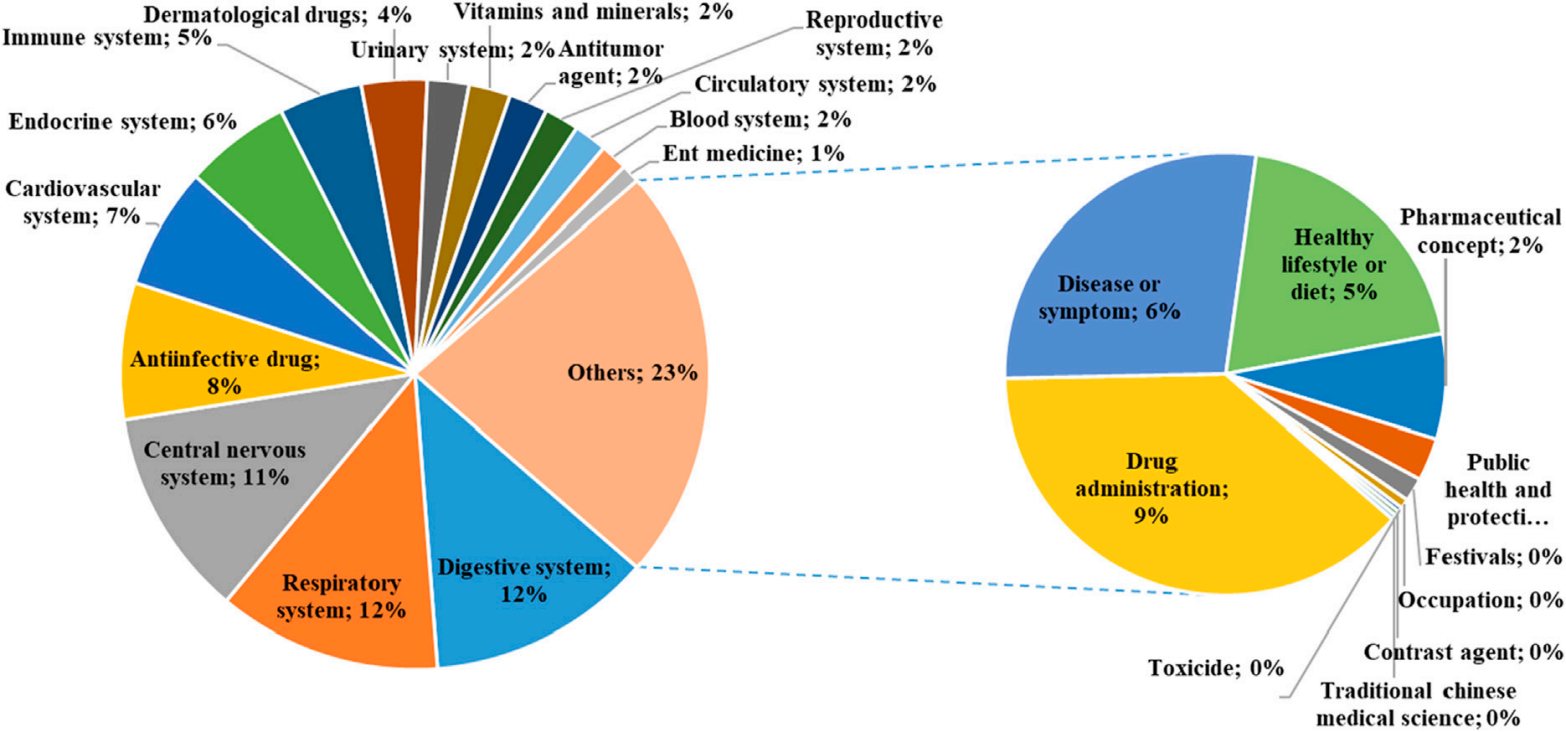
Theme distribution map of high-pageviews science popularization articles in 2015–2023.

The regional distribution of high-pageviews popular science articles of PSM Medicine Shield Public Welfare from 2015 to 2023 is shown in Figure 2. The theme distribution of high-pageviews popular science articles of PSM Medicine Shield Public Welfare from 2015 to 2023 is shown in Figure 3. Popular science articles involving drugs are classified and ranked by pharmacological effects as follows: digestive system (157 articles), respiratory system (155 articles), central nervous system (145 articles), anti-infective drugs (95 articles), cardiovascular system (84 articles), endocrine system (74 articles), immune system (58 articles), dermatological drugs (45 articles), urinary system (29 articles), vitamins and minerals (29 articles), anti-tumor drugs (27 articles), reproductive system (24 articles), circulatory system (23 articles), blood system (19 articles), and otorhinolaryngological drugs (13 articles). There were 287 articles on other topics, ranked as follows: drug management (110 articles), diseases or symptoms (79 articles), healthy lifestyle or diet (57 articles), medical concepts (22 articles), public health and protection (9 articles), festivals (5 articles), occupations (2 articles), antidotes (1 article), contrast agents (1 article), and traditional Chinese medicine (1 article).

### 3.2 High-frequency Keywords and word cloud diagram

After ranking the top 50 terms of the text TF-IDF values of 1,264 high-pageviews popular science articles, the word cloud diagram drawn based on the keywords and their frequencies is shown in Table 1. The word frequency data of some keywords are presented in Table 1. The top 25 keywords in order are: dosage, infection, efficacy, side effects, taking medicine, children, contraindication, popular science, COVID-19, skin, cough, prescription drugs, traditional Chinese medicine, virus, cold, stopping medication, drugs, use with caution, methods, pregnancy, seeking medical treatment, receptor, blood pressure, injection, Chinese patent medicine.

**TABLE 1:** Frequency table of the top 25 keywords.

Order	Keyword	Frequency
1	Dose	80
2	Medicine	69
3	Infect	68
4	Efficacy	65
5	Side effect	60
6	Take medicine	56
7	Popularization of science	56
8	Children	56
9	Forbidden	53
10	COVID-19	52
11	Skin	51
12	Prescription drug	51
13	Cough	50
14	Traditional Chinese medicine	49
15	Virus	47
16	Be used with caution	47
17	Catch a cold	47
18	Drug withdrawal	47
19	Practice	45
20	At once	43
21	Be pregnant	42
22	Go to a doctor	41
23	Blood pressure	41
24	Acceptor	40
25	Injection	40

### 3.3 Co-occurrence knowledge graph and time series analysis of keywords

**FIGURE 4:**
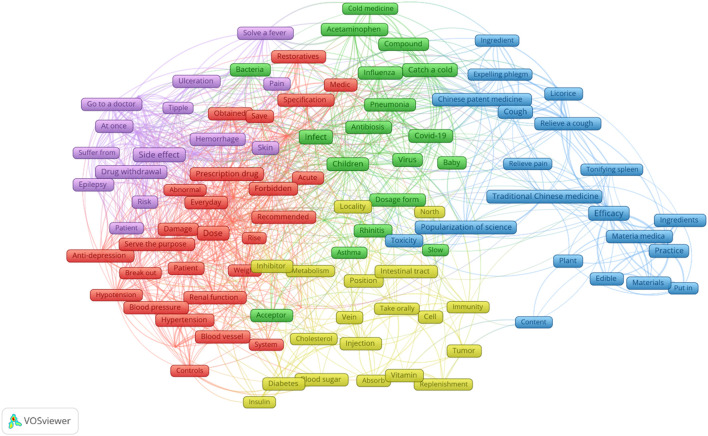
Keyword co-occurrence knowledge graph of high-pageviews science popularization articles in 2015–2023.

**FIGURE 5:**
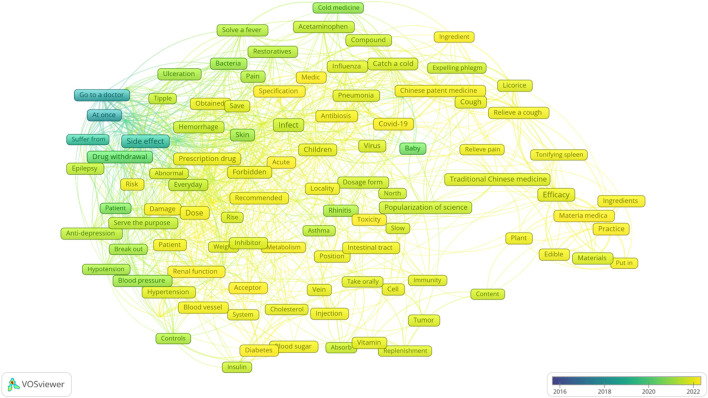
Keyword time series graph of high-pageviews science popularization articles in 2015–2023.

After calculating the TF-IDF values of the top 50 terms in each high-pageviews science popularization article published by PSM Medicine Shield Public Welfare from 2015 to 2023, the network relationship of the top 123 keywords was further analysed. The co-occurrence knowledge graph of these keywords is shown in Figure 4, which can be clustered into five categories. The time series analysis visualization of these keywords is shown in Figure 5. The top 123 keywords are mainly concentrated in the time period from 2020 to 2022. The five themes of clustering are drug dosage, drug side effects, paediatric respiratory infections, the efficacy of traditional Chinese medicine, and the administration route of the drugs.

### 3.4 Topic modeling with LDA

**FIGURE 6:**
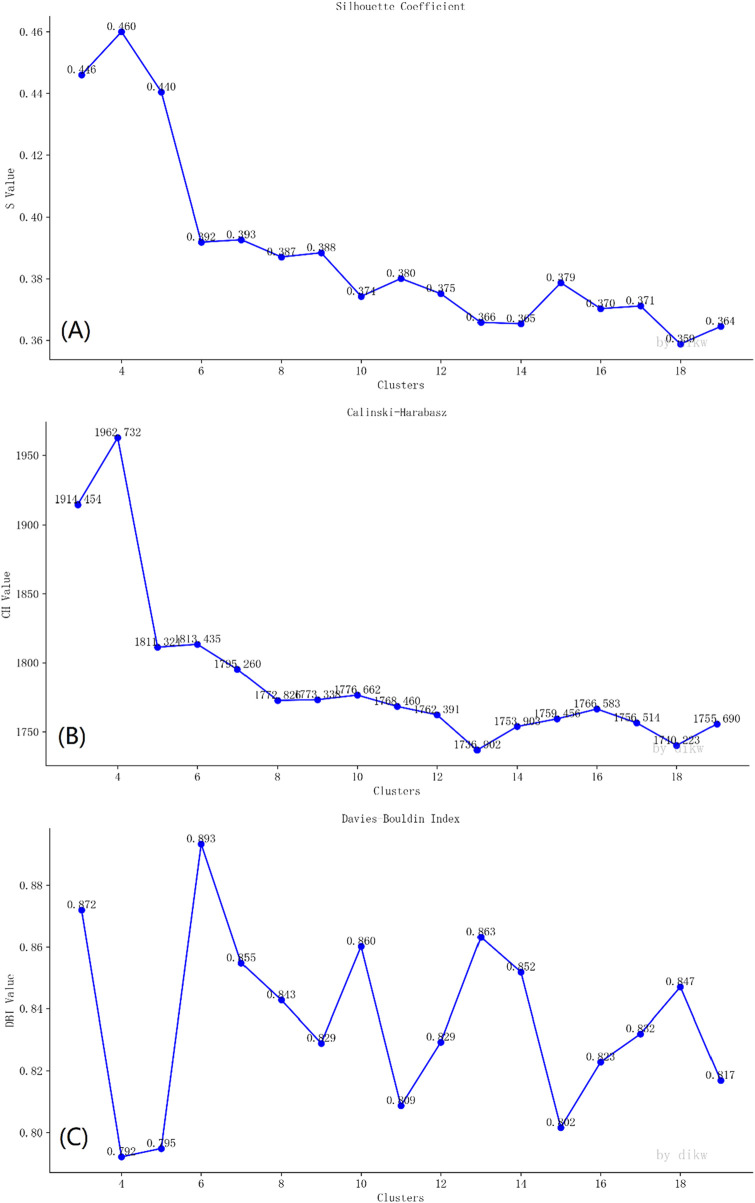
Determination of the number of clustering themes through **(A)** Silhouette coefficient, **(B)** Calinski-Harabasz index and **(C)** Davies-Bouldin index.

The optimal number of clusters was determined utilizing the Silhouette coefficient (Figure 6A), Calinski-Harabasz index (Figure 6B) and Davies-Bouldin index (Figure 6C). The largest Silhouette coefficient is 0.460 in 4 clusters. The largest Calinski-Harabasz index is 1962.732 in 4 clusters. The smallest Davies-Bouldin index is 0.792 in 4 clusters. This result indicates that when the theme is set to 4 categories, the clustering effect is the best. Furthermore, we conducted tests using LDA topic models with different numbers of topics (Supplementary Figures S2–S10). The results show that when the number of clusters is 4, the bubble charts of the four themes do not overlap (Supplementary Figure S4). This enables the themes to be distinguished from each other and ensures the maximum number of themes. This once again confirms that selecting 4 as the number of themes is appropriate.

**FIGURE 7:**
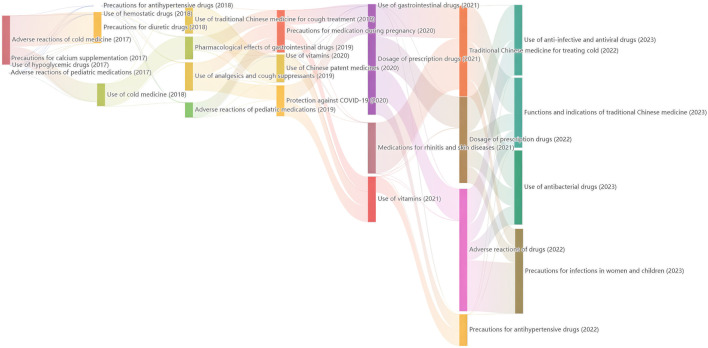
The trend of topic evolution based on LDA model from 2017 to 2023.

The top 10 keywords of the four topics under the LDA model were showed in Supplementary Table S1. The four topics are as follows. (1) Safe usage and risk warning of antipyretic and analgesic drugs. (2) The vital energy and blood regulation plan of traditional Chinese medicine for strengthening the spleen. (3) The safety management of the initial dosage of antidepressants and suicide risk. (4) A popular science introduction to the traditional Chinese medicine and antiviral solution for COVID-19. The dynamic changes in topics of pharmic science popularization are shown in a Sankey diagram (Figure 7). In 2017, the research on adverse reactions mainly focused on cold medicines. In 2022, the river width flowing from adverse reactions of drugs to precautions for infections in women and children was the greatest, indicating that the public is increasingly concerned about precautions for infections in children and women from adverse reactions of drugs. From the perspective of nodes, node “Traditional Chinese for treating cold” in 2022 is larger than node “Use of traditional Chinese medicine for cough pregnancy” in 2019. The usage of traditional Chinese medicine remains a hot topic, but the focus of attention has shifted from colds to medication during pregnancy.

### 3.5 Topic modeling with BERTopic model

**FIGURE 8:**
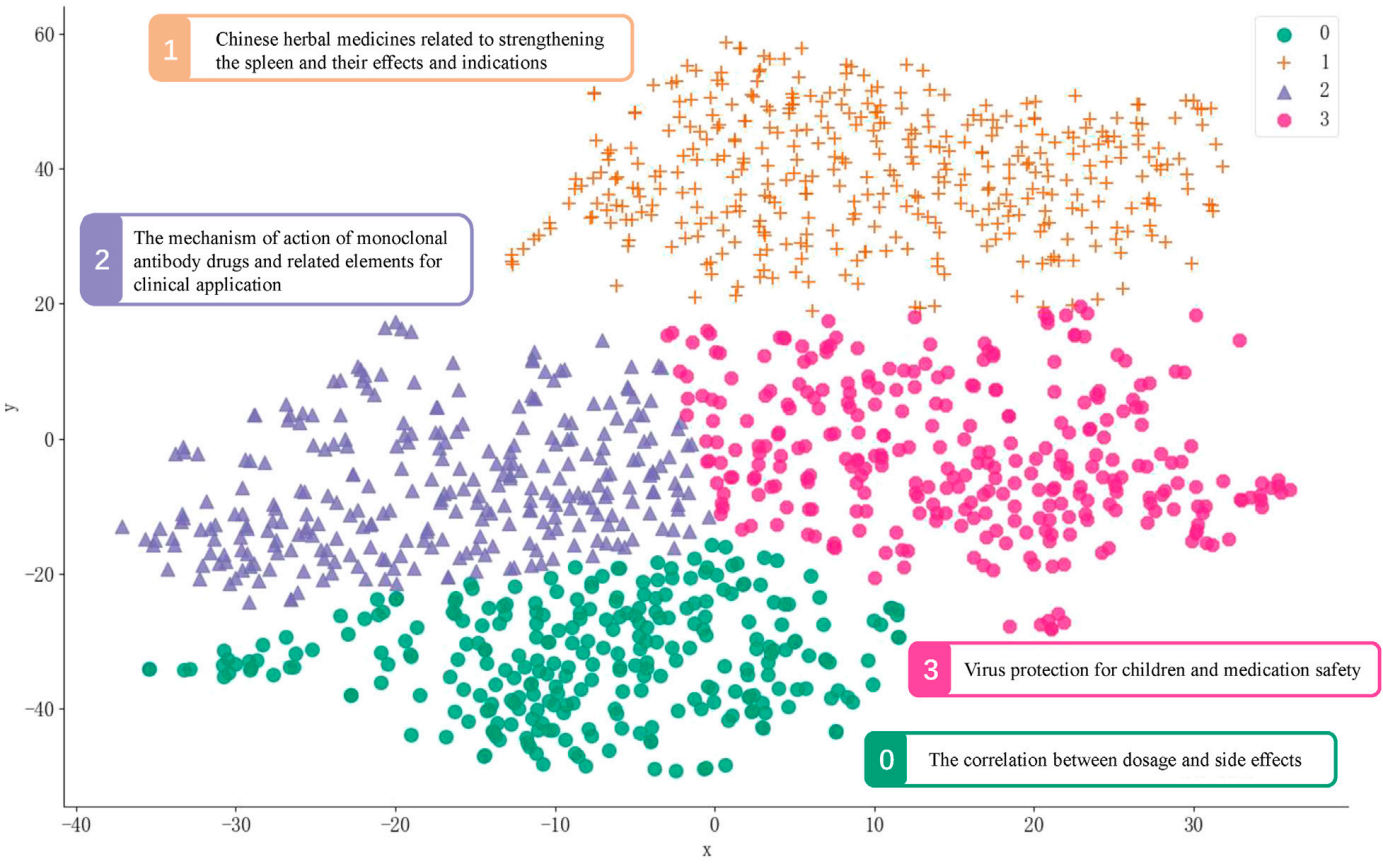
Document topic distribution chart based on BERTopic model.

We used Sentence-BERT for vectorized representation of multi-source text data to achieve unified representation and fusion of multi-source text data in the same semantic vector space. The two-dimensional vector distribution map of each text was shown in Figure 8. The top 10 keywords of the four topics based on BERTopic model and the topic representation employs the Log Likelihood Ratio (LLR) algorithm were shown in Supplementary Table S2. The four topics are “The correlation between dosage and side effects”, “Chinese herbal medicines related to strengthening the spleen and their effects and indications”, “The mechanism of action of monoclonal antibody drugs and related elements for clinical application” and “Virus protection for children and medication safety”.

## 4 Discussion

Pharmic science popularization, as a powerful supplement to offline activities, is playing an important role. PSM Medicine Shield Public Welfare, which is a non-profit organization jointly initiated and established by the China Association of Nonprescription Drugs, the Chinese Pharmaceutical Association and the Chinese Medical Association, has exerted an increasingly significant influence in science popularization activities in China. Given the emphasis of Chinese policies on science popularization activities, we conducted a retrospective analysis of the textual analysis of the science popularization efforts carried out by PSM Medicine Shield Public Welfare through the WeChat platform, which could help us understand the development of science popularization in China. The number of science popularization articles published by the “PSM Medicine Shield Public Welfare” WeChat official account has been increasing year by year from 2015 to 2023. The account released its first pharmic science popularization article on 28 November 2015. A total of 6,457 articles had been pushed by 31 December 2023. Among them, the number of articles released in 2022 was the highest, reaching 1,255. The proportion of high-pageviews articles reached its peak in 2022, accounting for 33.47% of the articles released that year. As shown in Figure 1, high-pageviews articles were mainly concentrated in 2021–2023, which reflects that the articles of this official account in these 3 years received more clicks, possibly related to the improvement of article quality and the increased public attention to pharmacology science popularization knowledge. From the perspective of regional distribution, the institutions that released high-pageviews articles were mainly concentrated in Guangdong Province, indicating that the main group of article publishers of this official account was in Guangdong Province. Compared with other cities in Guangdong Province, Guangzhou had the highest proportion of high-reading science popularization articles, suggesting that the online pharmacology science popularization work of medical institutions in Guangzhou was at the forefront of the entire Guangdong Province. From the perspective of the theme distribution of reading science popularization articles, drugs for the digestive system, respiratory system, and central nervous system ranked the top three. Among other high-pageviews science popularization articles, the category of drug management had the highest proportion, such as the storage management of drugs, the best time for taking drugs, the route of administration, the brewing methods of traditional Chinese medicine, family medicine boxes, and the medical insurance directory, indicating that the public also had a strong interest in this type of daily life-related drug knowledge.

Through the calculation of the TF-IDF algorithm, we selected the keywords of each text of these 1,264 high-pageviews science popularization articles and statistically analyzed the frequency of the terms appearing in these texts. The keyword word cloud is shown in Supplementary Figure S1. The larger the font size of the text, the higher the frequency of the term. From Supplementary Figure S1 and Table 1, we could find that the term “dose” appeared most frequently in high-reading articles, indicating that the public was particularly concerned about the popularization of drug dosage. In the keyword co-occurrence knowledge graph (Figure 4), different clusters are represented by different colors, and the size of the keywords represents the frequency of the theme’s appearance. The distance between the subjects represents the similarity of the themes. The closer the distance between two keywords, the more similar the themes.

The red region mostly relates to drug dose, particularly for prescription medications that impact kidney or blood pressure. The concern about the dosage of antihypertensive drugs by public may be due to the fact that hypertension is one of the chronic diseases with a high prevalence rate. On the other hand, the dosage should be adjusted or combination therapy should be initiated in a timely manner for patients whose blood pressure is not controlled after taking a low dose of a single drug. For patients who have grade 1 hypertension with low or moderate risk and systolic blood pressure of 140–149 mmHg, or elderly or frail patients, the dosage can be gradually increased to the full dose after starting with a low dose. The adverse reactions of antihypertensive drugs are often related to the dosage, such as hypokalemia caused by thiazide diuretics and ankle edema caused by calcium channel blockers. Optimal antihypertensive drug dose is difficult to determine (Dimmitt et al., 2019). In addition, after satisfactory control is achieved through treatment, the dosage of antihypertensive drugs can be gradually reduced to discontinuation, but direct discontinuation is not allowed, and blood pressure and treatment effects should be monitored regularly during the treatment process. The dosage of antihypertensive drugs is not fixed but adjusted dynamically based on blood pressure conditions, which is the reason why the public pays close attention to such health education.

The purple area is mainly related to the side effects of drugs. Keywords such as “taking medicine”, “stopping medicine”, “immediately”, and “seeking medical attention” are close to the central theme word “side effects” in this area, indicating that they are likely to appear in the same text and can be classified under the same theme. A survey found that 90.84% of the respondents were concerned about adverse reactions, and 70.24% hoped to obtain relevant knowledge about adverse reactions, especially how to reduce or avoid their occurrence and the measures to take when they occur (Gao et al., 2024). The high reading volume of the public on adverse drug reactions also reflects that the health education on drugs through public accounts has a certain positive effect on enhancing patients’ awareness of medication safety. For instance, through health education, the public learns that some mild adverse reactions can be reduced by adjusting the way of taking medicine, such as taking some drugs that stimulate the gastrointestinal tract after meals to reduce symptoms like nausea and vomiting. If the adverse reactions are severe, the medicine should be stopped immediately and medical attention sought. As shown in Figure 4, the public is more concerned about the adverse reactions of antiepileptic drugs. Due to the mechanism of antiepileptic drugs, long-term use, and drug interactions, the incidence of adverse reactions is relatively high, so the safety of antiepileptic drugs has received increasing attention. Common adverse reactions of antiepileptic drugs include drowsiness, memory loss, and unsteady gait, which can lead to poor patient compliance or even discontinuation of the medication, resulting in treatment failure. Currently, 50% of patients need combined treatment with antiepileptic drugs to control epileptic seizures, but the interactions between different drugs, such as carbamazepine, a liver enzyme inducer, can increase the metabolism of other drugs, reduce the efficacy of combined medication, and increase the risk of epileptic seizures in patients.

The green space mostly relates to the topic of infection, particularly frequent pediatric respiratory diseases like pneumonia and influenza. Due to the underdeveloped immune system and poor resistance in children, they are more prone to respiratory infections than adults. Since *mycoplasma* pneumonia has similar symptoms to the common cold or influenza, many parents initially confuse respiratory infections in children and give them antiviral or antibiotic drugs without proper diagnosis, not only failing to provide targeted treatment but also delaying the best treatment time. In terms of symptoms, influenza is mainly characterized by fever, accompanied by sore throat and body aches, while *mycoplasma* pneumonia is mainly dry cough, which may be accompanied by fever and other symptoms. Since influenza is caused by the influenza virus, antiviral drugs are needed for treatment; *mycoplasma* pneumonia is caused by *Mycoplasma* pneumoniae and is mainly treated with antibiotics. As shown in Figure 4, the public also shows a strong interest in health education related to common respiratory infections in children.

The blue area mainly introduces themes related to the efficacy of Chinese patent medicines and traditional Chinese medicine, mainly focusing on cough and expectorant Chinese patent medicines, as well as some Chinese medicines that can be used as food ingredients. In traditional Chinese medicine, cough is a disease characterized by the failure of the lung to disperse and descend, the upward movement of lung air, and the production of cough sounds or expectoration of phlegm. It is one of the main symptoms of lung diseases, and its causes are divided into two major categories: external invasion and internal injury. External cough is caused by the invasion of the six external pathogenic factors, while internal cough is caused by the dysfunction of internal organs. The two can influence and transform each other (Ma et al., 2022). The treatment methods are based on the main causes and mechanisms of cough, using the principle of syndrome differentiation and treatment. The core of Chinese patent medicines for treating cough is to transform phlegm and stop coughing, supplemented by heat-clearing, heat-reducing, and tonifying the lung and kidney. In addition to being interested in traditional Chinese medicine (TCM) and Chinese patent medicines for treating coughs, the public also pays attention to the concept of “food as medicine” and health preservation as a topic of conversation during leisure time. As a precious treasure of traditional Chinese culture, TCM health preservation can help regulate the functions of various systems in the human body, prevent and improve common diseases. However, the public should also be aware of the contraindications of TCM combinations.

The light-yellow area mainly focuses on the administration routes of drugs, such as oral and injection for diabetes medications. As a common chronic disease, diabetes requires long-term treatment. However, a single treatment method often fails to effectively control blood sugar. Studies have shown that the combination of long-acting insulin injection and oral hypoglycemic drugs can avoid the significant drop in blood sugar caused by a single insulin injection in a short period of time. The combination is more moderate, reduces the dosage, and has higher safety. Research has found that strengthening the education of patients on the safe injection of insulin can improve the accuracy of the injection site, method, time, and dosage, enhance the safety and effectiveness of medication, significantly reduce the incidence of adverse reactions, and improve patients’ treatment compliance (Wang and Luo, 2019). As shown in Figure 4, the public may have a certain interest in subcutaneous injection drugs that can be taken home. Generally, for subcutaneous injection drugs that can be taken home, the first injection is usually done in the hospital, but patients often cannot remember all the details at once. Compared with the oral administration route, the dosage and usage of subcutaneous injection are more complex, and patients’ compliance is poorer. Therefore, there is a greater need for medication science popularization on the usage and dosage of subcutaneous injection.

To visually observe the evolution of keywords in high-reading articles of PSM Drug Shield Public Welfare from 2015 to 2023, we used different colors to distinguish the years when the keywords appeared and drew a keyword time series chart for visualization analysis. As shown in Figure 5, between 2018 and 2020, the high-reading science popularization articles of this public account mainly focused on the theme of side effects. Between 2020 and 2021, the more prominent keywords were “children”, “babies”, “patients”, “blood pressure”, and “low blood pressure”. The vast majority of the remaining keywords mainly appeared after 2021. This indicates that the public’s demand for science popularization themes has changed, from initially focusing on the side effects of drugs to children’s medication, drug dosage, the efficacy of TCM and Chinese patent medicines, and drug administration routes. This may be related to the continuous improvement of the public’s pharmaceutical science popularization literacy, and their interest in science popularization has gradually shifted from understanding adverse drug reactions to learning about practical drug use problems encountered in daily life. We can draw the same conclusion from Sankey diagram based on LDA model.

Both LDA and BERTopic models generated meaningful topics from the case study on knowledge related to drugs, highlighting key areas of concern and daily medication relevance. The BERTopic model identified 4 distinct topics, emphasizing the safety of medication usage such as the correlation between dosage and side effects and virus protection for children and medication safety. This model also uncovered themes related to the pharmaceutical knowledge related to monoclonal antibodies and traditional Chinese medicine that the public is concerned about. In comparison, the LDA model generated 4 topics with a broader focus on traditional Chinese medicine enhances the function of the spleen and has therapeutic effects against the novel coronavirus. The LDA model also explored the safe usage of antipyretic, analgesic drugs and antidepressants. These results are consistent with those keywords of the co-located knowledge graph, such as drug dosage, drug side effects, paediatric respiratory infections and the efficacy of traditional Chinese medicine.

On operational strategy and recommendations of pharmic science popularization, we have the following suggestions. The title of an article is a reading guide, an important tool in determining whether the audience needs to read the details of the article. Through moderate exaggeration, suspense, digital display and other methods to draw up the title, can attract the attention of the audience to a certain extent, increase the click-through rate of the article, so as to enhance the communication effect. In addition, it is suggested that the WeChat public account operation team should insist on pushing scientific, high-quality and interesting health science articles at a fixed time, so that users can develop a good habit of continuous reading, enhance user stickiness, and cultivate a stable user group.

## 5 Conclusion

In order to accurately identify the public’s thematic reading habits and improve the communication effect of pharmic science popularization, this paper uses text mining technology for efficient analysis to help science popularization workers carry out science popularization operation and decision-making. This study conducted text mining based on the TF-IDF algorithm and used VOSviewer for keyword visualization analysis. It can efficiently and quickly mine the public’s interested pharmaceutical science popularization themes from existing public account platforms. It also demonstrated the effectiveness of both LDA and BERTopic in the text mining of pharmic science popularization. By combining the TF-IDF algorithm with NLP techniques, text vectorization can be achieved, thereby facilitating our understanding of the text’s theme. This method can help pharmacists establish a stronger connection with users, disseminate drug knowledge closely related to the public’s life, make the theme selection of science popularization articles more targeted, enhance the readability of science popularization articles and the influence of public accounts, and promote the high-quality development of pharmaceutical science popularization. Pharmaceutical workers can improve the health literacy of the public by improving the quality of science popularization, improving the communication efficiency, and disseminating more health knowledge to the public.

The advantage of this study is that through retrospective data analysis of the platform, it can understand the public’s demand for science popularization, which overcome the disadvantages of large sample collection and long-term traceability of questionnaire survey. We can more efficiently explore the public habits and needs of science popularization from the text. However, this method has the following drawbacks. Firstly, due to the timeliness of the hot spot, the duration of the hot spot is about 2 years from the time series analysis. It is recommended to collect the data again every year to better capture the topic and increase the number of clicks. Secondly, the study determines a good article only by the page view counts. It does not incorporate other engagement metrics, such as shares, comments, and likes. Thirdly, the findings are made from a single WeChat public account. This makes it difficult to generalize the identified topics to other platforms, demographic groups, or regions. In the future, more high-quality science popularization articles from various dimensions and more data from different public accounts will be included to achieve the wide promotion of this technology.

## Data Availability

The raw data supporting the conclusions of this article will be made available by the authors, without undue reservation.
