# Correction: Research on strategies for enhancing drug knowledge dissemination on Chinese social media WeChat public accounts based on text mining technology

**DOI:** 10.3389/fphar.2025.1694687

**Published:** 2025-09-05

**Authors:** Frontiers Production Office

**Affiliations:** Frontiers Media SA, Lausanne, Switzerland

**Keywords:** natural language processing, topic modelling, term frequency-inverse document frequency (TF-IDF), VOSviewer, WeChat, visualization analysis, pharmic science popularization, medication adherence

The affiliation of Reviewer Randy Cahya Wihandika was erroneously given as Brawijaya University Hospital, Indonesia. The correct affiliation is Brawijaya University, Indonesia.

The original article has been updated.

