# Supplementary material for: Research on strategies for enhancing drug knowledge dissemination on Chinese social media WeChat public accounts based on text mining technology
Source: Front Pharmacol. 2025 Aug 26;16:1569863. doi: 10.3389/fphar.2025.1569863 (PMC12446869; doi:10.3389/fphar.2025.1569863)
Supplement: Supplementary file 1 [file DataSheet1.docx]

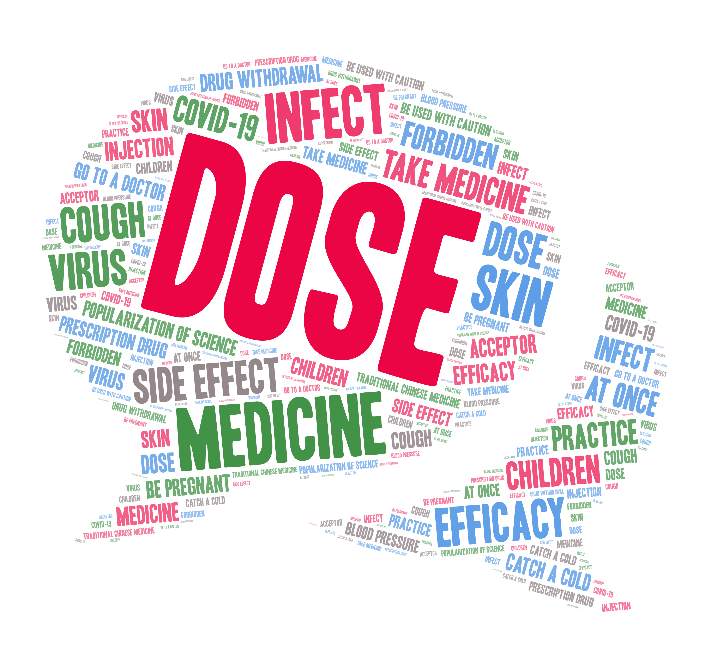


**Supplement Figure 1.** Word cloud map for the top 25 keywords in 2015-2023


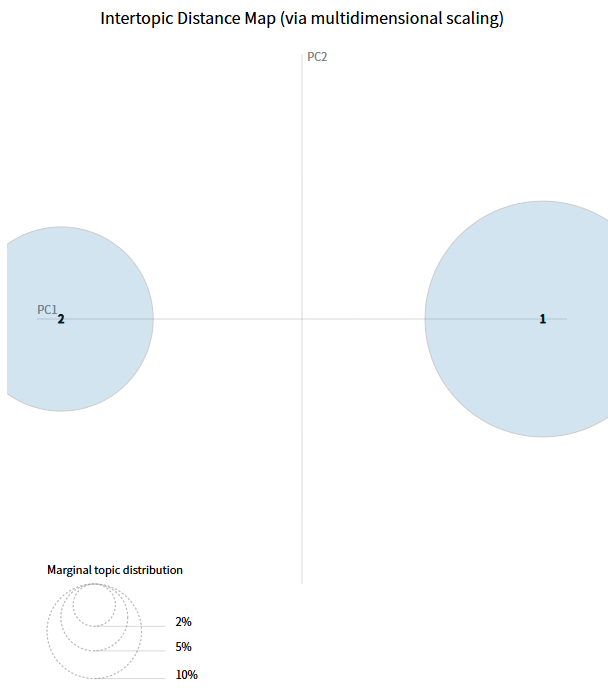


**Supplement Figure 2.** Intertopic distance map of LDA topic modeling with 2 topics.


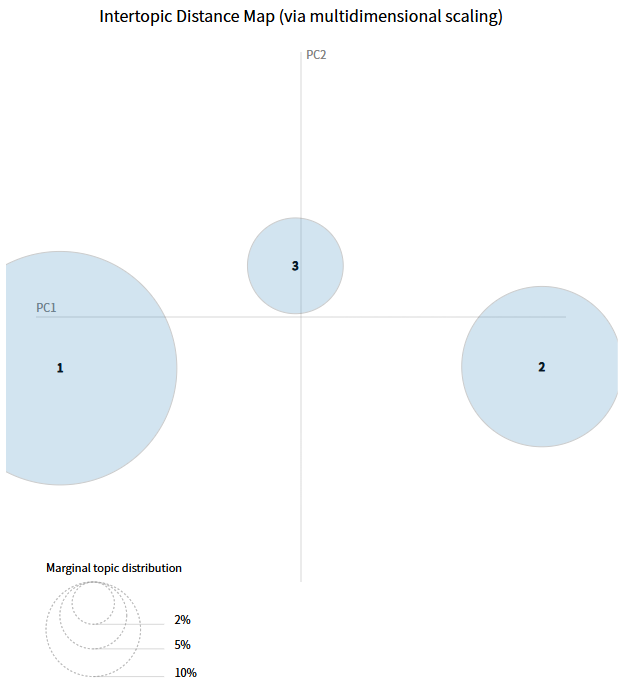


**Supplement Figure 3.** Intertopic distance map of LDA topic modeling with 3 topics.


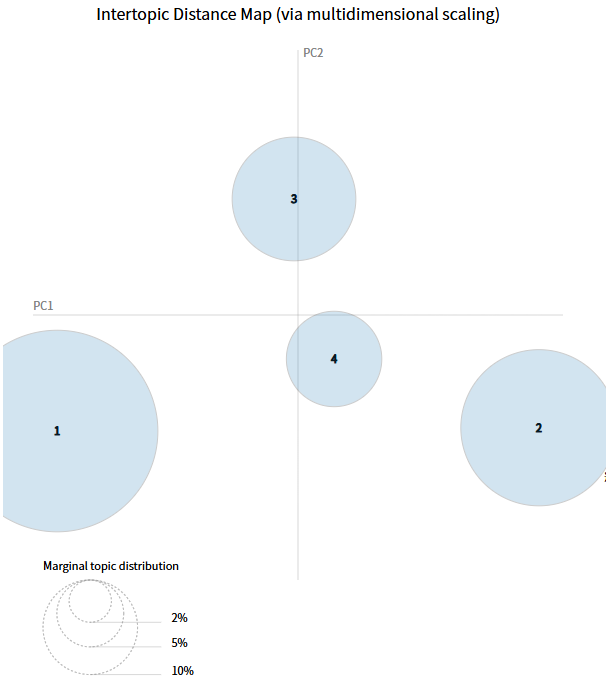


**Supplement Figure 4.** Intertopic distance map of LDA topic modeling with 4 topics.


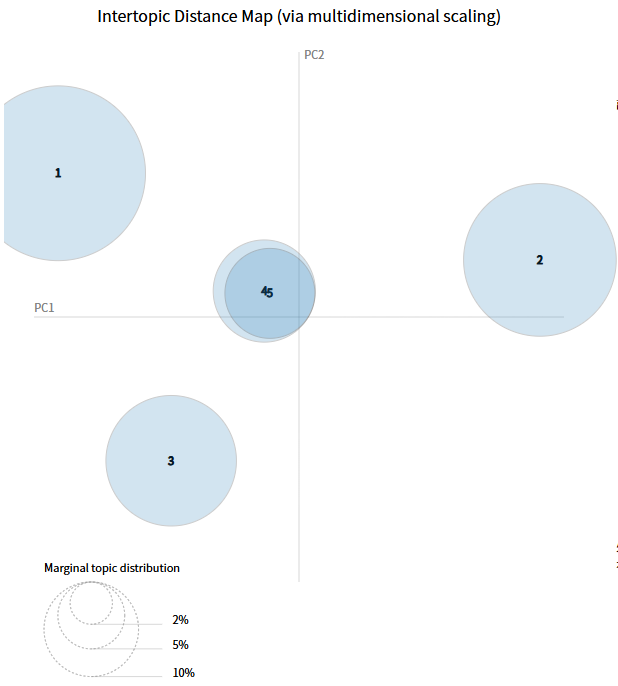


**Supplement Figure 5.** Intertopic distance map of LDA topic modeling with 5 topics.


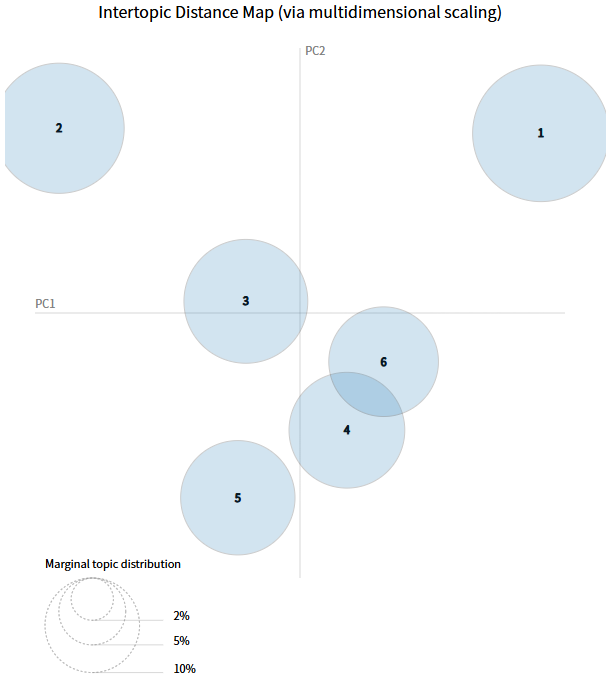


**Supplement Figure 6.** Intertopic distance map of LDA topic modeling with 6 topics.


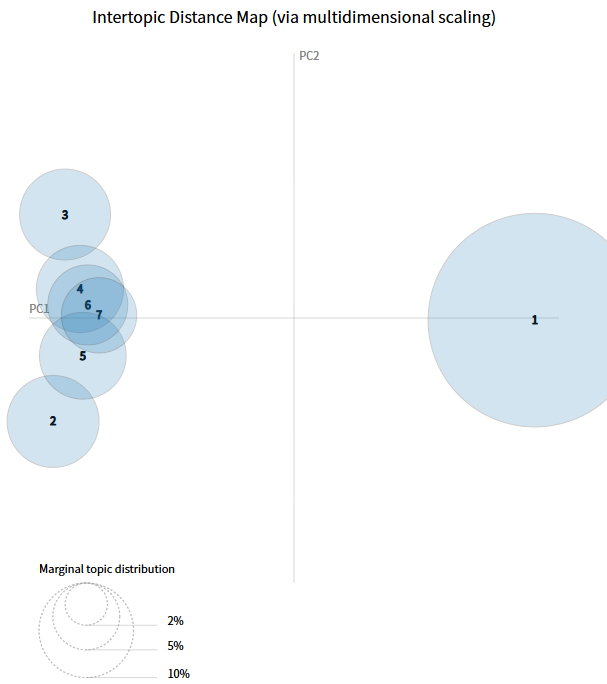


**Supplement Figure 7.** Intertopic distance map of LDA topic modeling with 7 topics.


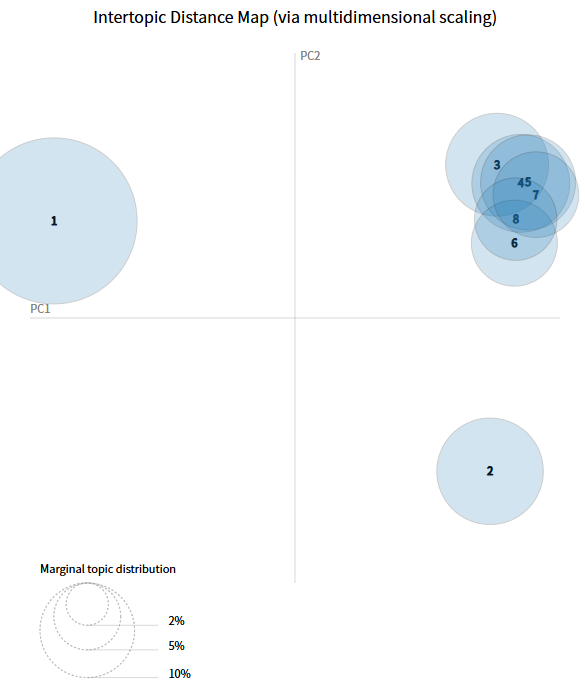


**Supplement Figure 8.** Intertopic distance map of LDA topic modeling with 8 topics.


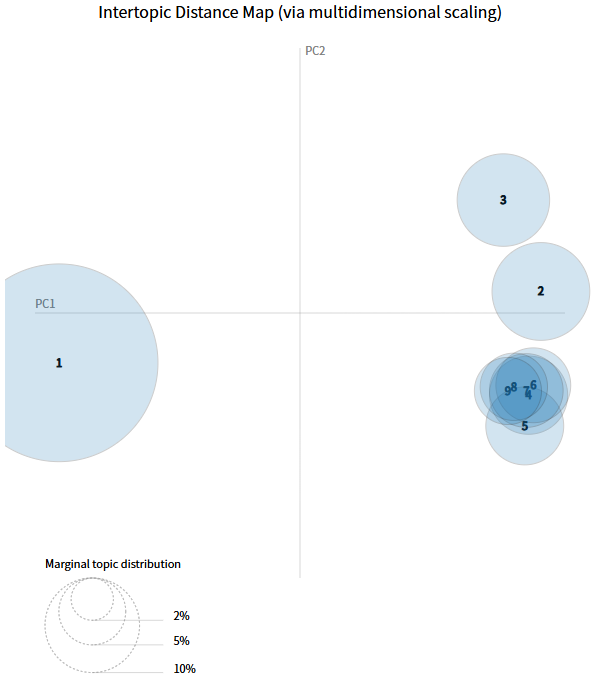


**Supplement Figure 9.** Intertopic distance map of LDA topic modeling with 9 topics.


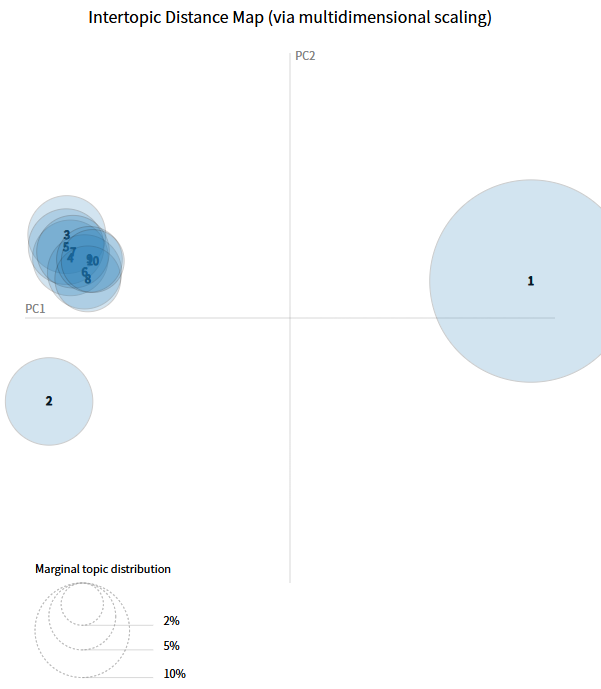


**Supplement Figure 10.** Intertopic distance map of LDA topic modeling with 10 topics.

**Supplement Table 1.** The top 10 keywords of the four topics based on LDA model

| Topic | Key words | Probability |
| --- | --- | --- |
| Safe usage and risk warning of antipyretic and analgesic drugs | Side effects | 0.001 |
|  | Skin | 0.001 |
|  | Immediately | 0.001 |
|  | Take medicine | 0.001 |
|  | Seek medical treatment | 0.001 |
|  | Infection | 0.001 |
|  | Doctor | 0.001 |
|  | Children | 0.001 |
|  | Bleeding | 0.001 |
|  | Antipyretic | 0.001 |
| The vital energy and blood regulation plan of traditional Chinese medicine for strengthening the spleen | Efficacy | 0.001 |
|  | Licorice | 0.001 |
|  | Spleen and stomach | 0.001 |
|  | Astragalus membranaceus | 0.001 |
|  | Method | 0.001 |
|  | Strengthen the spleen | 0.001 |
|  | Traditional Chinese medicine | 0.001 |
|  | Vital energy and blood | 0.001 |
|  | Poria cocos | 0.001 |
|  | Atractylodes macrocephala | 0.001 |
| The safety management of the initial dosage of antidepressants and suicide risk | Dosage | 0.001 |
|  | Anti-depression | 0.001 |
|  | Suicide | 0.001 |
|  | Mental illness | 0.001 |
|  | Obstacle | 0.001 |
|  | Depression | 0.001 |
|  | Drug | 0.001 |
|  | Behavior | 0.001 |
|  | Starting | 0.001 |
|  | Lower blood pressure | 0.001 |
| A popular science introduction to the traditional Chinese medicine and antiviral solution for COVID-19 | COVID-19 | 0.001 |
|  | Concentration | 0.001 |
|  | Virus | 0.001 |
|  | Lower blood sugar | 0.001 |
|  | Coronavirus | 0.001 |
|  | Popular science | 0.001 |
|  | Solution | 0.001 |
|  | South | 0.001 |
|  | Traditional Chinese medicine | 0.001 |
|  | Rhizome | 0.001 |

**Supplement Table 2.** The top 10 keywords of the four topics based on BERTopic model and the topic representation employs the Log Likelihood Ratio (LLR) algorithm.

| Topic | Key words | LLR value |
| --- | --- | --- |
| The correlation between dosage and side effects | Dosage | 190.6137 |
|  | Side effects | 164.7535 |
|  | Antidepressant | 142.9201 |
|  | Seek medical attention | 129.4918 |
|  | Blood pressure | 127.205 |
|  | Anxiety | 125.4172 |
|  | Discontinue medication | 122.0996 |
|  | Concomitant use | 121.8454 |
|  | Hypotension | 121.1797 |
|  | Patient | 106.2562 |
| Chinese herbal medicines related to strengthening the spleen and their effects and indications | Functions | 285.8037 |
|  | Preparation method | 215.3909 |
|  | Materials | 162.7398 |
|  | Edible | 155.2205 |
|  | Licorice | 151.6585 |
|  | Herbal medicine | 143.5939 |
|  | Strengthen the spleen | 129.2345 |
|  | Main treatment | 119.6616 |
|  | Spleen and stomach | 114.8751 |
|  | Dried tangerine peel | 114.8751 |
| The mechanism of action of monoclonal antibody drugs and related elements for clinical application | Medication | 124.8511 |
|  | Receptor | 102.0369 |
|  | Infection | 101.2329 |
|  | Inhibitor | 87.42647 |
|  | Maintain | 74.03645 |
|  | Injection | 73.56756 |
|  | Histamine | 67.56268 |
|  | Blood drug concentration | 65.91542 |
|  | Antibody | 64.97931 |
|  | Acetaminophen | 62.51336 |
| Virus protection for children and medication safety | Virus | 123.3469 |
|  | Local | 101.9749 |
|  | Medicine | 72.62833 |
|  | Nasal cavity | 68.24279 |
|  | Children | 68.24279 |
|  | Antibiotics | 66.53087 |
|  | Contact | 66.08454 |
|  | Area | 65.53945 |
|  | Safety | 56.86899 |
|  | WeChat | 56.86899 |
